# Effects of *Bacillus altitudinis* inoculants on cigar tobacco leaf fermentation

**DOI:** 10.3389/fbioe.2024.1417601

**Published:** 2024-07-09

**Authors:** Wen Song, Xi Chen, Jun Yu, Jingyu Qiao, Jinpeng Yang, Xiong Chen, Zhi Wang

**Affiliations:** ^1^ Key Laboratory of Fermentation Engineering (Ministry of Education), Cooperative Innovation Center of Industrial Fermentation (Ministry of Education and Hubei Province), Hubei University of Technology, Wuhan, China; ^2^ Hubei Institute of Tobacco Science, Wuhan, China

**Keywords:** cigar tobacco leaf, fermentation, *Bacillus altitudinis* inoculants, aroma substance, metagenomics and metabolomics

## Abstract

**Introduction:**

Microbial succession and metabolic adjustment during cigar tobacco leaf (CTL) fermentation are key factors to improve the quality and flavor of CTLs. However, the interactions in the above processes remain to be further elucidated.

**Methods:**

*Bacillus altitudinis* inoculants were added to the CTLs, and metagenomics and metabolomics were used to analyze the effects of the inoculants on regulating microbial succession, metabolic shift, and aroma production during fermentation.

**Results and discussion:**

The addition of the inoculants reinforced the CTL macromolecule transformation and facilitated the aroma production efficiently, and the total aroma production was increased by 43% compared with natural fermentation. The omics analysis showed that *Staphylococcus* was a main contributor to fatty acid degradation, inositol phosphate metabolism, energy supply (oxidative phosphorylation), nutrient transport (ABC transporter and phosphotransferase system [PTS]), and aroma production (terpenoid backbone biosynthesis, phenylalanine metabolism, and degradation of aromatic compounds). Furthermore, *Staphylococcus* was positively correlated with TCA cycle intermediates (citric acid, fumaric acid, and aconitic acid), cell wall components, peptidoglycan intermediates (GlcNAc-1-P and UDP-GlcNAc), and phytic acid degradation products (inositol). The characteristics collectively showed *Staphylococcus* to be the most dominant in the microbial community at the genus level during microflora succession. The addition of the inoculants supplemented the nutritional components of the CTLs, enhanced the metabolic activity and diversity of bacteria such as *Corynebacterium*, improved their competitive advantages in the microflora succession, and facilitated the richness of microbial communities. Additionally, a metabolic shift in nicotine degradation and NAD + anabolism from *Staphylococcus* to *Corynebacterium* in fermentation with inoculants was first observed. Meanwhile, the significantly correlative differential metabolites with *Staphylococcus* and *Corynebacterium* were a metabolic complement, thus forming a completely dynamic fermentation ecosystem. The results provided evidence for CTL fermentation optimization.

## 1 Introduction

Biological macromolecules such as starch, cellulose, proteins, and alkaloids in cigar tobacco leaves (CTLs) can be degraded and converted into aroma substances by fermentation (Jiang et al., 2021; Jia et al., 2023), producing a unique flavor and taste (Zhang et al., 2020). The traditional fermentation process of CTLs is significantly influenced by environmental factors (Li et al., 2020). Meanwhile, cigar styles are also influenced by CTL-producing areas and the local climate (Zheng et al., 2022). The China cigar market is developing rapidly (He et al., 2023); however, there is still a gap in flavor and quality (Cai et al., 2019) compared with the cigars produced in Cuba, Brazil, and Dominica.

The addition of microorganisms and nutrients during CTL fermentation can improve the tobacco quality (Hu et al., 2022; Wen et al., 2023). CTLs treated with *Bacillus subtilis* could reduce the starch and cellulose contents and improve the CTL quality (Dai et al., 2020). The addition of a cocoa medium to the CTL fermentation process highlighted the CTL coffee aromas and enhanced the content of carotenoid degradation products (Zong et al., 2023). The addition of *Filobasidium magnum* and *Bacillus kochii* at a ratio of 3:1 could reduce the irritation and increase the sweetness and CTL aroma (Wu et al., 2021). The addition of *Fritillaria cirrhosa* extract, loquat extract, and glutinous rice wine to CTLs also increased aroma component production such as phenylethanol and solanone (Cai et al., 2019).

CTL fermentation is a mixed solid-state fermentation system with multiple microbial communities. Omics analysis is an effective tool for elucidating fermentation mechanisms (Wu et al., 2023). Huang et al. investigated the influence of the addition of *Bacillus subtilis* on CTL fermentation and found that *Pantoea* was the dominant bacterium, and phenylalanine metabolism was the main pathway affecting the aroma components (Huang et al., 2022). Zhang et al. analyzed the relationship between the microbial community and metabolites in aged tobacco leaves and found that *Pantoea* and *Pseudomonas* were the dominant genera, and differential metabolites were mainly enriched in pathways such as fatty acid metabolism (Zhang et al., 2022).

Our previous study found that, as an essential nutrient involved in the cellular metabolic process (Vance et al., 2003), the soluble phosphate was limited in the CTL fermentation process, and *B. altitudinis* in CTL fermentation hydrolyzed insoluble phosphates and enhanced the water-soluble phosphate content and aroma substance yield (Qiao et al., 2023). However, the interactions between the microbial community succession and the metabolism of aroma substances during fermentation were not yet clear. Therefore, using metagenomic and metabolomics analyses, the effects of *B. altitudinis* inoculants on the diversity and structural adjustment of the microbial community, metabolic pathway shift, and aroma component synthesis were investigated during CTL fermentation, providing evidence for the scientific regulation of CTL fermentation.

## 2 Materials and methods

### 2.1 Materials

The CTLs were CX14 after air drying in Enshi, Hubei Province, China. All the chemical reagents used in the study were purchased from Sinopharm Group, China.

*B. altitudinis* was isolated from the surface of cigar core CX14 and stored in the laboratory.

The bacterial culture (LB) medium consisted of 10 g/L peptone, 10 g/L NaCl, 5 g/L yeast extract powder, and the initial medium, pH = 7.2∼7.4, and was autoclaved at 121°C for 20 min.

The washing buffer consisted of 15.76 g/L Tris-HCl, 18.612 g/L EDTA-Na_2_, 81.9 g/L NaCl, 20 g/L polyvinylpyrrolidone (PVP), 1 g/L Tween-20, and the initial medium, pH = 8.0.

The extraction solution consisted of 0.02 g/L L-2-chlorophenylalanine and CH_3_OH:H_2_O = 4:1 (v:v).

### 2.2 Methods

#### 2.2.1 Preparation of *B. altitudinis* inoculants

*B. altitudinis* in a solid LB medium was inoculated in a shake flask with 50 mL LB medium using an inoculation ring. The shake flask with the medium was cultured at 37°C and 200 r/min for 24 h. The obtained seed solution was centrifuged at 4°C and 12,000 r/min for 5 min, the supernatant was discarded to collect wet cells, and then, the wet cells were resuspended in sterile deionized water. The operation was repeated three times. Then, it was mixed with glucose, glutamate, (NH_4_)_2_HPO_4_, and water to prepare *B. altitudinis* inoculants. The amount of *B. altitudinis*, glucose, sodium glutamate, and (NH_4_)_2_HPO_4_ added to the CTLs was 7.5 × 10^11^ CFU/kg (Qiao et al., 2023), 20 g/kg, 2.0 g/kg, and 2.0 g/kg, respectively.

#### 2.2.2 CTL fermentation

A handful of CTLs was untied and then was scattered. Afterward, the prepared microbial inoculants or water (the control) was evenly sprayed on the surface of CTLs. After the water was naturally absorbed, the moisture content in different parts of the CTLs was measured. When the moisture contents were about 30%, the water content in the CTLs reached a balance state. Then, 80 kg CTLs were stacked and loaded into a fermentation box, and then, the fermentation box was placed in the fermentation room. The temperature and humidity in the room were controlled at 30°C and 80%, respectively.

CTLs were stacked as follows: the CTL petioles were outward and were stacked layer by layer in the fermentation box (120 cm × 80 cm × 80 cm). A cotton cloth and a box cover were placed on the upper layer of the stacked CTLs.

Box fermentation cycles were conducted as follows: a temperature sensor was placed in the CTL core to show the temperature in the stacked core during the fermentation process. When the CTL stack temperature increased to a maximum value, then decreased, and remained constant at a certain temperature for approximately 1 day, the CTL stacks were turned over, and the fermentation process was continued for approximately 8 days. Then, the CTLs were restacked, and the second fermentation process was started. After the next 8 days or so, the second pile-turning was conducted, and then, the CTLs were stacked again, and the third fermentation process was carried out. After the third 8 days or so, the third fermentation was done, and then, the third pile-turning was conducted. In the previous study, it was determined that the CTL aroma content of the third pile-turning sample was the highest, so it was determined that fermentation was completed after the third pile-turning.

CTL samples were obtained as follows: when the CTL stacks were turned over, 1 kg of CTLs was randomly extracted from the core of the pile. The sample before fermentation was named CK_0. The samples of the first, second, and third pile-turning of natural fermentation were named CK_1, CK_2, and CK_3, respectively. The samples of the first, second, and third pile-turning of microbial inoculant fermentation were named BA_1, BA_2, and BA_3, respectively.

#### 2.2.3 Extraction and determination of the volatile aroma substances

The samples were processed using a simultaneous distillation–extraction (SDE) technique, and volatile aroma substances were analyzed by GC-MS (Li et al., 2020).

The process was conducted under chromatographic conditions (Yi et al., 2023) using an HP-5MS capillary column (30 m × 0.25 mm × 0.25 μm). Helium was used as the carrier gas at a linear velocity of 1 mL/min. The transmission line temperature was set at 250°C, and the ion source temperature was set at 230°C. The oven temperature was programmed from 40°C (2 min), increasing at 2°C/min to 200°C, and maintained for 5 min at 10°C/min to 280°C. The mass spectra were recorded in the electron impact (EI) ionization mode at 70 eV and a scan mass range of 35–550 m/z. The peak identification of the target compounds was compared based on the National Institute of Standards and Technology database (NIST14).

#### 2.2.4 DNA extraction and metagenomic sequencing

Samples (10.0 g) were suspended in 100 mL washing buffer and subjected to ultrasound for 30 min; then, the microbial samples were obtained by filtrating the washing buffer using eight layers of gauze. Then, the supernatant was centrifuged (10,000 × *g* for 10 min), and the cells were obtained. Total genomic DNA of the sediment was extracted using the NEXTFLEX™ Rapid DNA-Seq Kit. The extracted DNA was checked using 1% agarose gels. Metagenomic libraries of the samples were obtained using the Illumina MiSeq platform. Raw sequence data were generated. Clean data for subsequent gene function analysis were obtained by filtering the raw data. CD-HIT was used to build a non-redundant gene catalog, and MetaGene software was used for open reading frame (ORF) prediction. A taxonomic assignment was carried out by using BLASTP alignment against the integrated non-redundant (NR) database of the National Center for Biotechnology Information (http://blast. NCBI. NLM. NIH. gov/blast. CGI). In addition, the resulting genes were annotated and classified according to species and function. All these analyses of raw data were performed using the Majorbio Cloud platform (https://cloud.majorbio.com).

#### 2.2.5 Metabolites and LC-MS/MS analysis

Samples (100 mg) were taken in a 2-mL centrifuge tube, and 6-mm grinding beads and 400 µL extract were added. After mixing, the samples were ground in a high-throughput tissue crusher (6 min, −10°C, 50 Hz). The samples were then extracted in a low-temperature ultrasonic bath for 30 min (5°C, 40 kHz) and then left to stand at −20°C for 30 min. The samples were centrifuged using a high-speed refrigerated centrifuge (15 min, 4°C, 13,000 × g). An aliquot of supernatant (350 µL) was dried under nitrogen and then redissolved (100 μL, isopropanol/acetonitrile = 1:1 v/v). The solution was vortexed for 30 s and then sonicated for 5 min at 40 KHz in an ice-water bath. The solution was centrifuged for 10 min (13,000 × g, 4°C); then, an aliquot of supernatant (20 µL) from each sample was mixed to generate a quality control (QC) sample, and all the samples were analyzed by UHPLC-MS/MS at Majorbio Bio-Pharm Technology Co., Ltd. (Shanghai, China). The LC-MS raw data were imported into Progenesis QI metabolomics processing software (Waters Corporation, Milford, United States) to obtain the data matrix. The MS and MS/MS mass spectrometry information was matched with the Meiji self-built database to obtain the metabolite information.

#### 2.2.6 Data processing and analysis

The experimental data were presented as the mean from at least triplicate experiments for each condition. R v4.0.0 was used to generate the heatmap. Origin 2019b and Excel 2021 software were used for data processing and analysis.

## 3 Results and analysis

### 3.1 Effects of microbial inoculants on the volatile aroma production of CTLs

**FIGURE 1:**
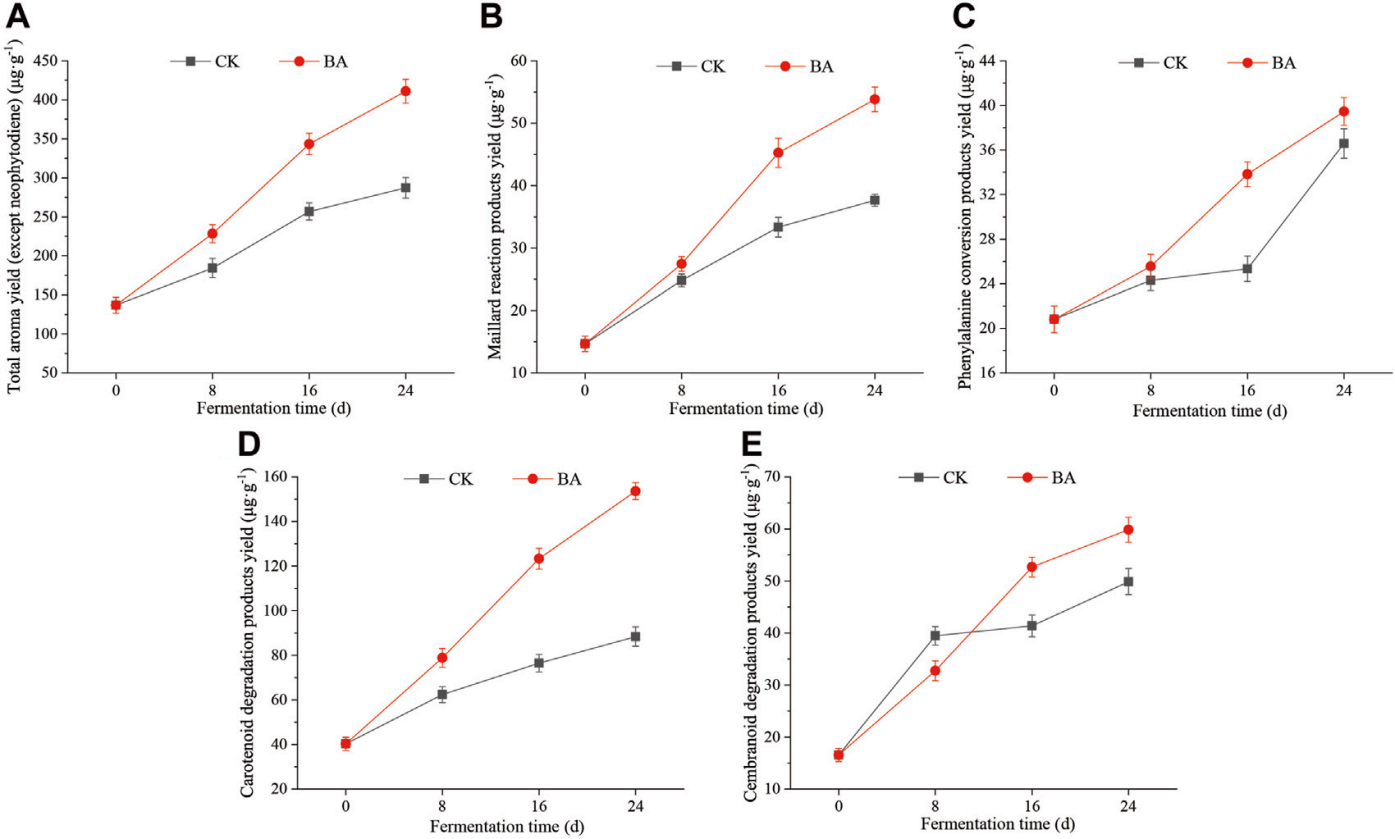
Effects of microbial inoculants on aroma production of CTLs. **(A)** Total aroma yield, **(B)** Maillard reaction product yield, **(C)** phenylalanine conversion product yield, **(D)** carotenoid degradation products yield, and **(E)** cembranoid degradation product yield.

In the process of fermentation, microbial communities utilize the nutrients of CTLs such as starch, cellulose, protein, carotenoids, and chlorophyll, for growth and metabolism, as well as aroma substance formation (Ma and Zengi, 2010). The aroma substances in CTLs can be divided into phenylalanine conversion products, carotenoid degradation products, Maillard reaction products, chlorophyll conversion products, and cembranoid degradation products. Neophytadiene, the degradation product of chlorophyll in CTLs, is considered to be the carrier of aroma substances (Yao et al., 2017), and so, it was not reckoned in the total aroma production (TAP). TAP in the fermentation process is shown in Figure 1A.

The TAP in CTLs before fermentation was 136.87 μg/g (CK_0), and it increased during the fermentation process. The TAPs of the first, second, and third pile-turning of the control sample were 184.28 μg/g, 256.78 μg/g, and 287.22 μg/g, respectively, and that of the microbial inoculant samples of the first, second, and third pile-turning was 228.32 μg/g, 343.45 μg/g, and 411.09 μg/g, respectively. The addition of microbial inoculants significantly facilitated the aroma production efficiency, and the TAPs during the same period were enhanced by 23.90%∼43.00% compared with those of the control.

The Maillard reaction products, such as furfural and furfuryl alcohol, have a kind of caramel aroma and can improve the taste of cigars and the aroma quality of CTLs (Seeman et al., 2003). The content of Maillard reaction products in CTLs before fermentation was 14.67 μg/g (CK_0), and it continued to increase during the fermentation process. The content in the first, second, and third pile-turning of the control sample was 24.81 μg/g, 33.34 μg/g, and 37.65 μg/g, respectively, and those of the microbial inoculant samples were 27.46 μg/g, 45.25 μg/g, and 53.82 μg/g, respectively, which were enhanced by 10.68%∼42.95% compared with the control.

During fermentation, the protein in CTLs can be hydrolyzed into various free amino acids, including phenylalanine. Then, phenylalanine can be converted to cherry-flavored benzaldehyde, rose-flavored phenylethanol, and flower-flavored phenylacetaldehyde by the microbial communities. These molecules also belong to the phenylalanine conversion products (Lei et al., 2015), and they make the cigar aftertaste more mellow (Yang et al., 2005; Ye et al., 2013). The content of phenylalanine conversion products gradually increased from 20.82 μg/g (CK_0), and the content in the first, second, and third pile-turning of the control sample was 24.31 μg/g, 25.33 μg/g, and 36.58 μg/g, respectively, and those of the microbial inoculant samples were 25.56 μg/g, 33.82 μg/g, and 39.46 μg/g, respectively, which were enhanced by 5.14%∼33.52% compared with the control.

Carotenoid degradation products directly affect the aroma type of CTLs, such as floral-flavored β-damascenone and dry fruit-flavored 4,7,9-megastigmatrien-3-one (Popova et al., 2019; Popova et al., 2020). The content of carotenoid degradation products in CTLs before fermentation was 40.25 μg/g (CK_0), and the content in the control sample in the first, second, and third pile-turning was 62.39 μg/g, 76.46 μg/g, and 88.38 μg/g, respectively. Those of the microbial inoculant samples were 78.83 μg/g, 123.32 μg/g, and 153.62 μg/g, respectively, which were enhanced by 3.10%∼73.82% compared with the control.

Solanone, a degradation product of cembranoids, has a carrot-like aroma and licorice flavor, which can make the aroma more mellow (Yun, 2016). The content of cembranoid degradation products in CTLs before fermentation was 16.54 μg/g (CK_0), and it continued to increase during the fermentation process. The content in the control samples in the second and third pile-turning was 41.38 μg/g and 49.87 μg/g, respectively. Those of the microbial inoculant samples were 52.68 μg/g and 59.84 μg/g, respectively, which were enhanced by 27.31% and 20% compared with the control, respectively.

### 3.2 Effects of microbial inoculants on the microbial composition of CTLs

**FIGURE 2:**
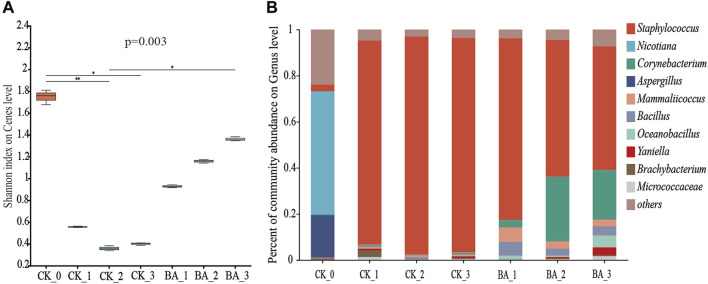
Effect of microbial inoculants on the microbial composition of CTLs. **(A)** α diversity Shannon index at the genus level and **(B)** columnar diagram of the genus horizontal community.

The microbial diversity can be reflected by the Shannon index, and the greater the value, the higher the microbial diversity. As shown in Figure 2A, the Shannon index before fermentation was 1.76, and that of the first, second, and third pile-turning of the CK was 0.55, 0.35, and 0.41, respectively. Meanwhile, that of the BA was 0.93, 1.16, and 1.36, respectively, which was increased by 69.09%∼231.71% compared with the CK. Although the microbial diversity of CTLs after fermentation was significantly decreased, the microbial diversity of the BA was significantly higher than that of CK, showing that the inoculants improved the microbial richness during the fermentation process.

Influences of microbial inoculants on the microbial community succession during the fermentation process are shown in Figure 2B. The top three genera in the microbial communities of CTLs before fermentation were *Nicotiana*, *Aspergillus*, and *Staphylococcus*, and their relative abundances accounted for 53.44%, 18.42%, and 2.94%, respectively. As the fermentation proceeded, the abundances of *Nicotiana* and *Aspergillus* in the first, second, and third pile-turning samples of the CK were significantly decreased by more than 300-fold, while that of *Staphylococcus* was 88.54%, 94.72%, and 93.11%, respectively, indicating that *Staphylococcus* was the absolute dominant genus. At the same time, *Staphylococcus* also became a dominant genus in the BA fermentation process, and the relative abundances in the first, second, and third pile-turning were 78.96%, 59.35%, and 53.69%, respectively.

After fermentation, the top three genera in the microbial communities of CK_3 were *Staphylococcus*, *Yaniella*, and *Corynebacterium*, and the relative abundances were accounted for 93.11%, 0.71%, and 0.68%, respectively. Those of BA_3 were *Staphylococcus*, *Corynebacterium*, and *Oceanobacillus*, and the relative abundances accounted for 53.69%, 21.29%, and 5.11%, respectively. Although the relative abundance of *Staphylococcus* was only 57.6% in the control, the proportion of *Corynebacterium* increased by 30.3 times, indicating that the microbial inoculants could effectively enhance the community richness and improve the metabolic diversity and fermentation efficiency. It also showed that *Corynebacterium* played an important role in the fermentation and aroma production of CTLs.

### 3.3 Effects of inoculants on microbial metabolism in the fermentation process

**FIGURE 3:**
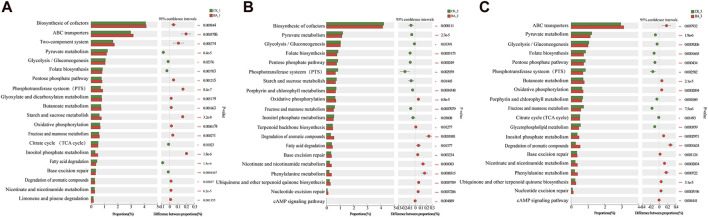
Difference test of metabolic pathways among groups. **(A–C)** Analysis of functional differences between the microbial group and the control group in the three-turning process. The ordinate represents the KEGG functional name at different classification levels, the abscissa represents the percentage value of a certain KEGG functional abundance of the sample, and different colors represent different groups. 0.01<*p* < 0.05*, 0.001<*p* < 0.01**, and *p* < 0.001***.

The addition of microbial inoculants changed the nutritional system and microflora structure of CTLs, and 430 significantly different metabolic pathways were observed by the metagenomic analysis. Here, the metabolic pathways related to carbon metabolism, energy metabolism, amino acid metabolism, and aroma substance metabolism were selected for the difference analysis (Figure 3).

As shown in Figure 3, during the first fermentation (fermentation time from the beginning to the first pile-turning, BA_1 vs. CK_1), the biosynthesis of cofactors (*p* < 0.01); nutrient transport (ABC transporter and phosphotransferase system); metabolism of starch, sucrose, fructose, and mannose; and oxidative phosphorylation process were vigorous (*p* < 0.001). It reflected that inoculants improved the growth and metabolic efficiency of the microbial communities. The rich nutrient environment led to a significant decrease in the degradation of delayed carbon sources such as fatty acids (BA_1, *p* < 0.001) and promoted the degradation efficiencies of carotenoid and aromatic substances such as limonene and pinene (BA_1, *p* < 0.05). The results were consistent with those obtained by Franzino et al. (2022) and Banozic et al. (2020). Therefore, the aroma production efficiency of BA_1 was 11.43 µg/g d, which was 1.92 times that of CK_1 (5.95 µg/g d) (Figure 1A). Additionally, significantly active nicotinate and nicotinamide metabolism in the inoculant-rich fermentation (BA_1, *p* < 0.001, Figure 3) was observed. It should be noted that nicotine degradation products were in the same metabolic pathway as the syntheses of nicotinate, nicotinamide, and nicotinamide adenine dinucleotide (NAD^+^) (Li et al., 2021), and so, nicotine continuously transformed in CTLs to nicotinate, nicotinamide, and NAD^+^ in BA_1 had a positive effect on the microflora energy metabolism and cigar products. Meanwhile, although phosphate was added to the inoculants, the inositol phosphate metabolism pathway was still significantly upregulated (*p* < 0.001), indicating that the supply of phosphate might be still a limiting factor in the system after the first fermentation.

During the second fermentation (time from the first pile-turning to the second pile-turning, BA_2 vs. CK_2), the metabolic pathways of carbon metabolism (starch, sucrose, fructose, and mannose) and nutrient transport (phosphotransferase system) were significantly downregulated (*p* < 0.05), and the cAMP signaling pathway was significantly upregulated (*p* < 0.01). The results suggested the system was in a carbon-limited state during the stage. So, as a reserved carbon source, fatty acid degradation metabolism was significantly enhanced (BA_2, *p* < 0.05) with improved oxidative phosphorylation efficiency (BA_2, *p* < 0.001). Additionally, as a component of the oxidative phosphorylation (Latimer et al., 2021), significantly upregulated biosynthesis of ubiquinone and terpenoid quinone was observed, which also played an active role in energy metabolism of BA_2 (*p* < 0.001). Meanwhile, nicotinate and nicotinamide metabolism was still significantly upregulated (BA_2, *p* < 0.001), which indicated that inoculants promoted the continuous transformation of nicotine. Moreover, inoculants reinforced the degradation metabolism of carotenoids and cembranoids (*p* < 0.05), as well as phenylalanine metabolism (*p* < 0.001), and the content of phenylalanine conversion products of BA_2 was increased by 33.52% compared with CK_2. The aroma production efficiency of BA_2 was 14.39 µg/g d, which was 1.59 times that of CK_2 (9.06 µg/g d) and 1.26 times that of BA_1 (Figure 1A).

During the third fermentation (time from the second pile-turning to the third pile-turning, BA_3 vs. CK_3), fructose and mannose metabolism, glycerolipid metabolism, and the phosphotransferase system were significantly decreased (*p* < 0.01) with a significantly upregulated cAMP signaling pathway (*p* < 0.01), which was related to an aggravated carbon-limited state of the system. Meanwhile, ubiquinone and terpenoid quinone biosynthesis; nicotinate and nicotinamide metabolism; oxidative phosphorylation; and the transformations of carotenoids, chlorophyll, nicotine, cembranoids, and phenylalanine were continuously enhanced (BA_3, *p* < 0.001). The aroma production efficiency of BA_3 was 8.46 µg/g d, which was 2.22 times that of CK_3 (3.81 µg/g d) (Figure 1A) and was only 59% of BA_2, indicating that the second fermentation period was the main stage of aroma production.

During the fermentation process, carbon metabolism and nutrient transport gradually decreased. Microbial inoculants could enhance the metabolic intensity, including the energy supply; transform CTL nutrients such as carotenoids, chlorophyll, and nicotine; and improve aroma component production.

### 3.4 Effects of the microbial genus on the metabolic contribution rate

**FIGURE 4:**
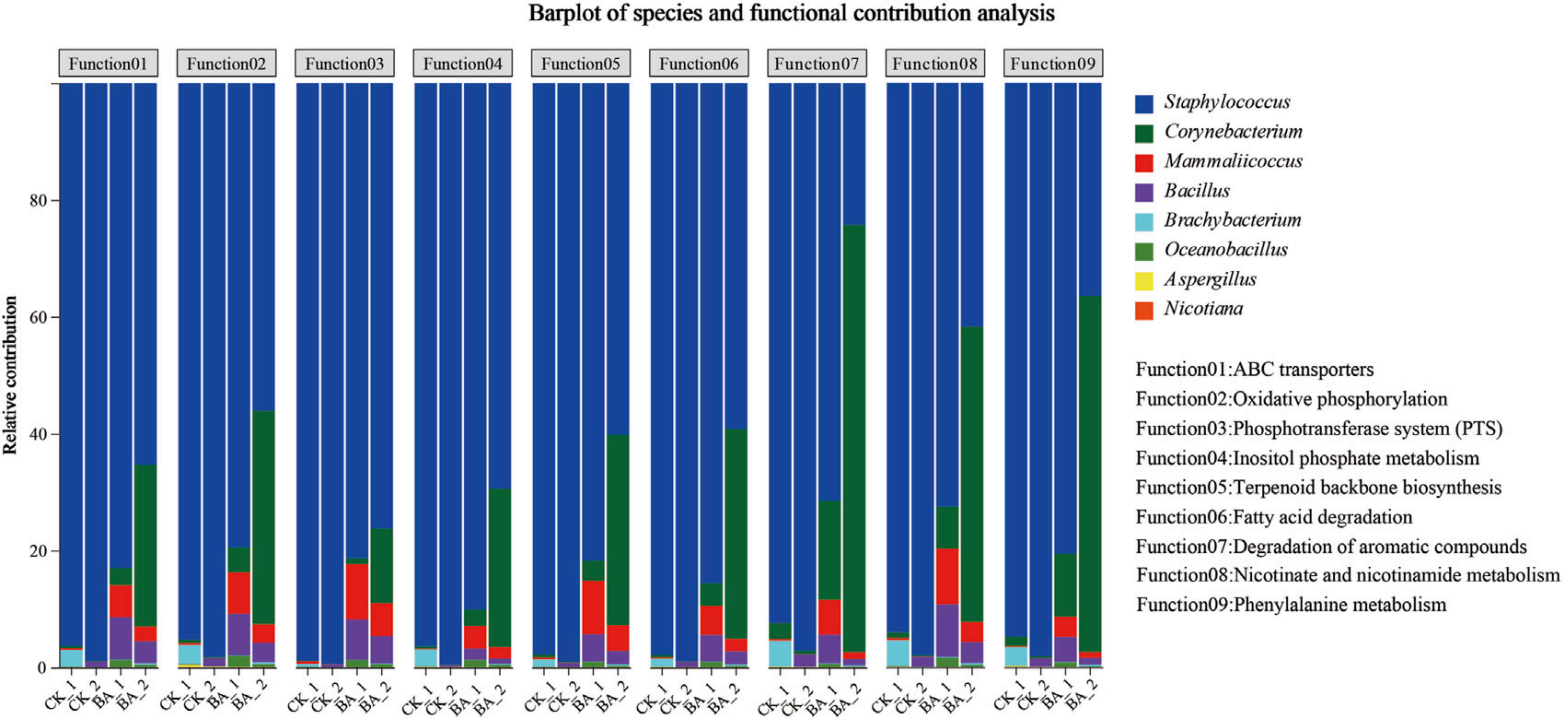
Effects of the microbial genus on the metabolism contribution rate.

The aroma production efficiency reached the highest during the second fermentation (time from the first pile-turning to the second pile-turning; Figure 1). So the effects of the microbial genus on the metabolic function contribution rate were analyzed during the stage (Figure 4).

In the figure, the abscissa is the CTL sample, and the ordinate is a metabolic contribution rate. The figure shows the contribution rate of different microbial genera to different functions.

Contribution rates of *Staphylococcus* in carbon metabolism (fatty acid degradation), inositol phosphate metabolism, energy supply (oxidative phosphorylation), nutrient transport (ABC transporters and phosphotransferase system), aroma production (terpenoid backbone biosynthesis, phenylalanine metabolism, and degradation of aromatic compounds), and nicotine degradation (nicotinate and nicotinamide metabolism) accounted for 92.38%∼98.84% in CK_1. Furthermore, those of *Staphylococcus* in CK_2 gradually increased up to 97.16%∼99.56%. These metabolic advantages promoted *Staphylococcus* to be the absolute dominant genus (Figure 2B). Meanwhile, during the second fermentation, contribution rates of *Corynebacterium* and *Mammaliicoccus* to the above pathways decreased from 0.11%∼2.71% (CK_1) and 0.09%∼0.41% (CK_1) to 0.01%∼0.57% (CK_2) and 0.01%∼0.06% (CK_2), respectively.

In the BA group, the contribution rates of *Staphylococcus* in BA_1 to the above metabolic pathways accounted for 71.47%∼90.10%, while those of *Corynebacterium*, *Mammaliicoccus*, and *Bacillus* were 0.99%∼16.90%, 3.48%∼9.54%, and 1.92%∼9.03%, respectively. During the second fermentation, contribution rates of *Staphylococcus* in BA_2 to carbon metabolism, energy supply, and nutrient transport were 56.07%∼76.22%, which decreased by 23%–42% compared with those of CK_2 (97.16%∼99.56%). The results suggested that the addition of inoculants promoted microflora richness and promoted metabolic diversity. Meanwhile, the contribution rates of *Mammaliicoccus* and *Bacillus* in BA_2 to the above metabolic pathways decreased to 0.54%∼5.61% and 0.02%∼4.72%, respectively. The highest metabolism of phenylalanine, aromatic compounds, nicotinate, and nicotinamide was shown by *Corynebacterium* (BA_2), and the contribution rates accounted for 60.93%, 73.09%, and 50.49%, respectively (Figure 4), which were associated with an increased *Corynebacterium* proportion in microbial communities (Figure 2B). Accordingly, the contribution rates of *Staphylococcus* to the above pathways were 36.47%, 24.28%, and 41.72% (BA_2; Figure 4), respectively, which were only 60%, 33%, and 83% of those of *Corynebacterium*.

The results showed that the addition of inoculants enhanced the nutritional components of the CTLs, provoked the bacterial metabolic activity, such as that of *Corynebacterium*, and improved their competitive advantages in the microflora succession and facilitated the richness and metabolic diversity of microbial communities.

### 3.5 Effects of microbial inoculants on differential metabolites after fermentation

**FIGURE 5:**
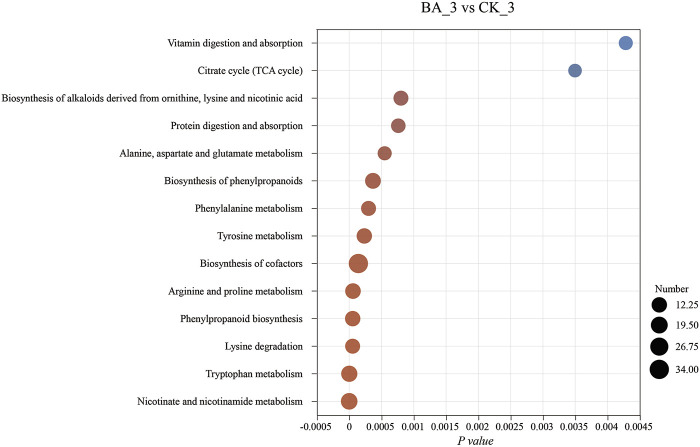
Effects of microbial inoculants on differential metabolite pathway enrichment. The abscissa is the enrichment significance, *p*-value. The ordinate is the KEGG pathway. The size of the bubbles in the figure represents the amount of compounds enriched in the pathway.

Microbial inoculants promoted the microflora succession by affecting nutritional structures of the system, which, in turn, affected the shifts in metabolic pathways and could be reflected by differential metabolites. As its aroma substance content was the highest at the end of fermentation (Figure 1), differential metabolites at the third pile-turning (BA_3 vs. CK_3) were analyzed. The differential metabolites were enriched in 266 metabolic pathways and are shown in Figure 5.

**FIGURE 6:**
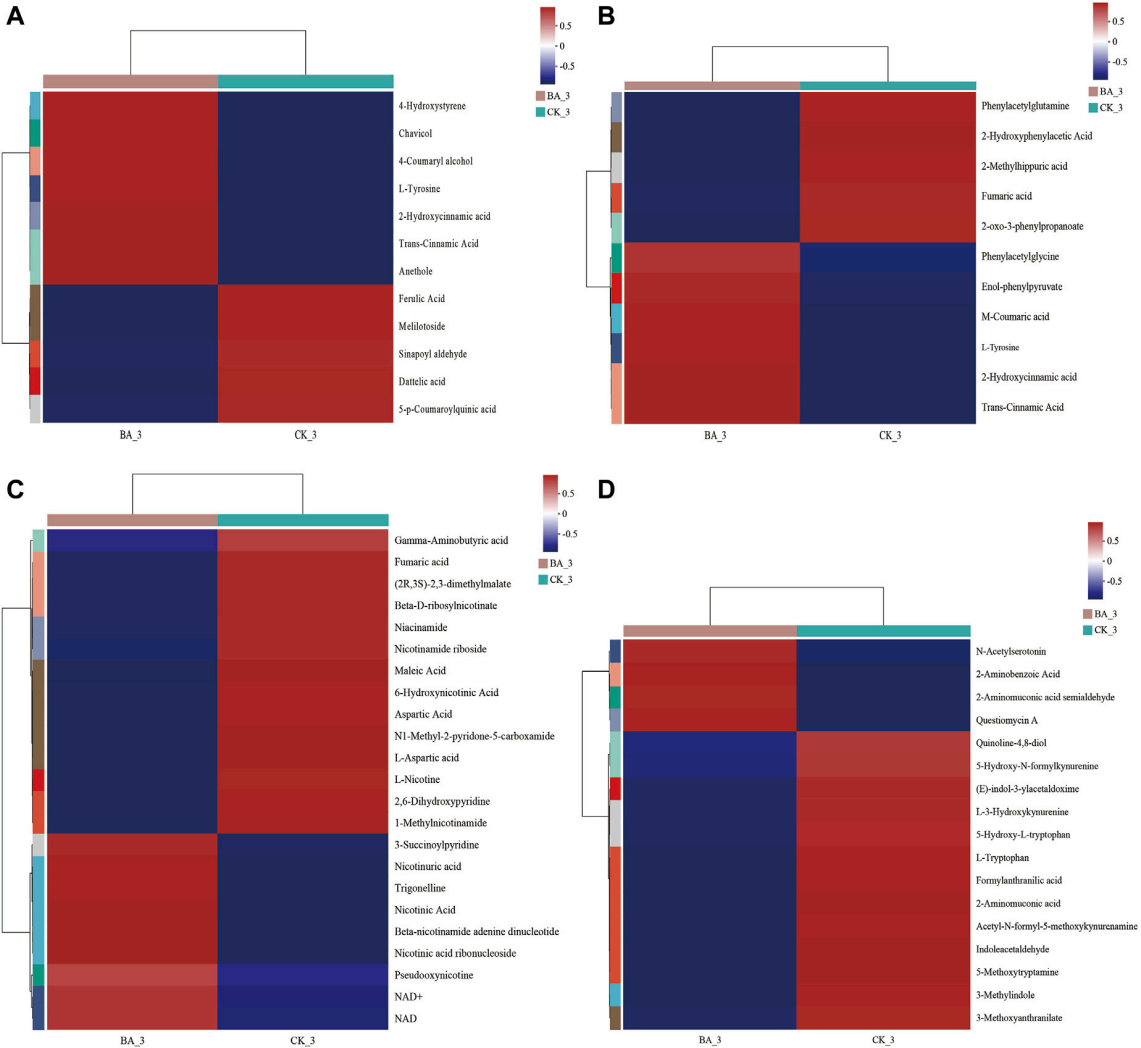
Effects of microbial inoculants on differential metabolites in metabolisms of phenylpropanoid **(A)**, phenylalanine **(B)**, nicotinate, nicotinamide **(C)**, and tryptophan **(D)**. A color-coded scale from blue to red indicates the amount of metabolites from low to high.

Metabolites in phenylalanine metabolism and phenylpropanoid biosynthesis of BA_3 were significantly upregulated (*p* < 0.001), including floral-flavored chavicol, honey-flavored cinnamic acid, and licorice-flavored anethole (Figure 6A). Moreover, milky-flavored hydroxycinnamic acid in phenylalanine metabolism was also significantly upregulated. The metabolites played a positive role in maintaining the quality and stability of cigar products for their antioxidant (Han et al., 2017) and antibacterial (Kuwahara et al., 2004; Abd et al., 2015) functions.

Nicotine, a nitrogen-containing alkaloid in CTLs, can be converted to nicotinate, nicotinamide, NAD^+^, and metabolites such as 3-succinoyl pyridine, 2,6-dihydroxypyridine, fumaric acid, and γ-aminobutyric acid through pyridine and pyrrole pathways (Chen et al., 2008; Yu et al., 2015; Li et al., 2017). In other words, these metabolites could be reused by microflora communities for growth and metabolism. Indeed, the contents of nicotine and its above-mentioned degradation products in BA_3 were lower than those in CK_3 (Figure 6C). The results suggested the nicotine tolerance and transformation performance in BA were enhanced, and nicotine could be continuously transformed into nicotinate, which promoted nicotinate and nicotinamide metabolism in the BA group during fermentation (*p* < 0.001; Figure 3). Subsequently, intermediate metabolites in nicotinate, nicotinamide, and tryptophan metabolism were significantly upregulated (Figure 6D) in BA_3, which significantly promoted NAD^+^ synthesis (Figure 6C) and oxidative phosphorylation (*p* < 0.001; Figure 3), thus increasing the biodiversity of BA_3 (BA_3 vs. CK_3; Figure 2).

### 3.6 Correlation characteristics of microorganisms and differential metabolites after fermentation

**FIGURE 7:**
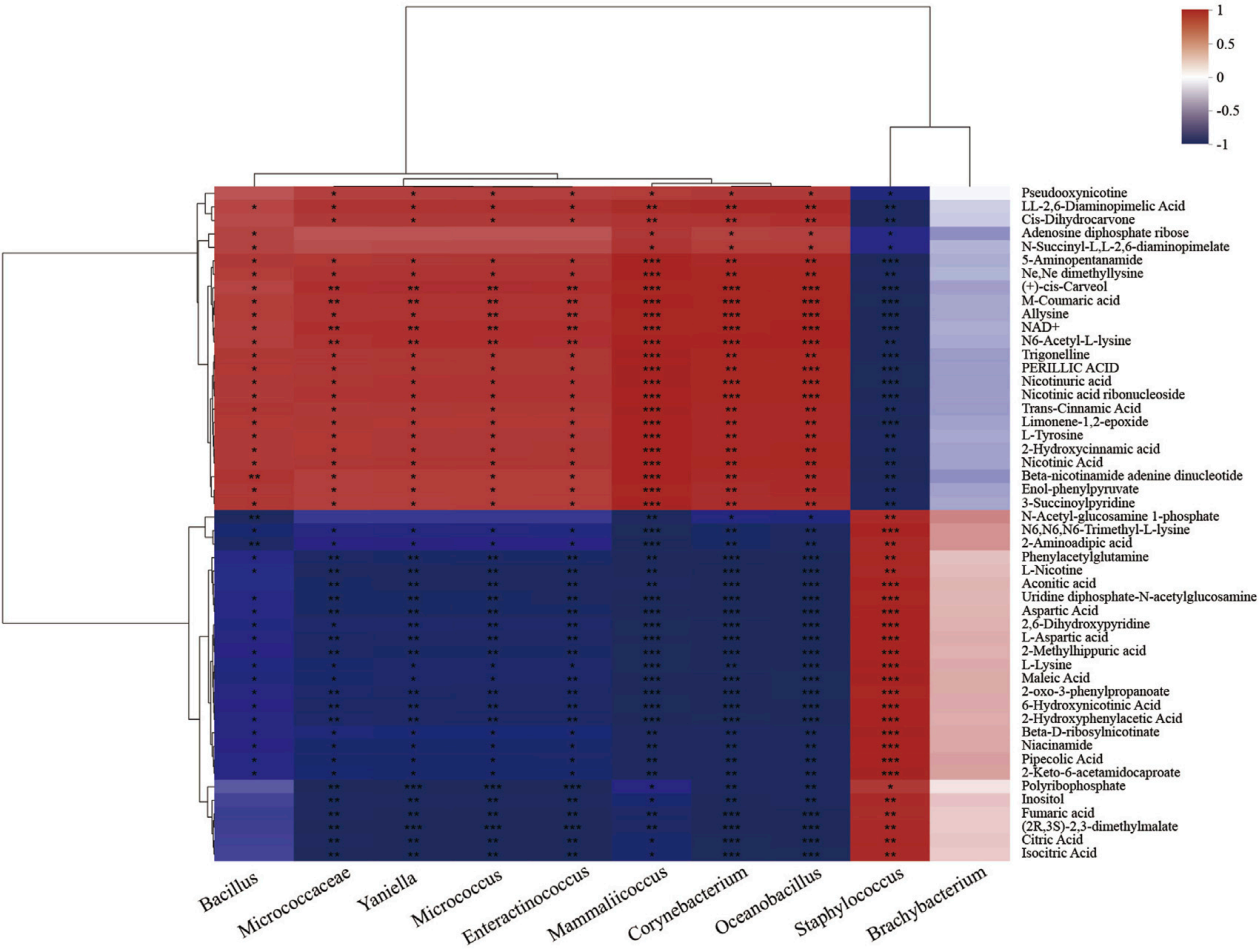
Heatmap of the correlation analysis between microorganisms and differential metabolites. Each grid represents the correlation between two attributes (metabolites and genus-level microorganisms), and different colors represent the size of the correlation coefficient between attributes.

Correlation analysis between microorganisms and intracellular differential metabolites after fermentation showed that *Staphylococcus* was positively correlated with citric acid, fumaric acid, L-nicotine, aconitic acid, N-acetyl-glucosamine-1-phosphate (GlcNAc-1-P), uridine diphosphate-N-acetyl-glucosamine (UDP-GlcNAc), and inositol (*p* < 0.05). Among them (Figure 7), citric acid, fumaric acid, and aconitic acid were the intermediate products of the TCA cycle (Engel et al., 2008; Li et al., 2020), indicating that *Staphylococcus* was the primary completer of energy metabolism, which was consistent with its maximum contribution rate in oxidative phosphorylation (Figure 4). In addition, as intermediates in the bacterial peptidoglycan biosynthesis, an effective supply of GlcNAc-1-P and UDP-GlcNAc was prerequisite for normal cell growth (Li et al., 2011). Both of them were positively correlated with *Staphylococcus* (Figure 6), which may be one of the reasons why *Staphylococcus* became the most dominant genus during fermentation.

Inositol was a product of phytic acid dephosphorylation (Karmakar et al., 2020; Li et al., 2022); the conversion was significantly upregulated during fermentation (Figure 3), indicating that the system might be in a phosphate-limited state. Inositol and inositol phosphate metabolism (Figure 4) was positively correlated with *Staphylococcus* (*p* < 0.01; Figure 7), thereby promoting the dephosphorylation of phytic acid and accelerating *Staphylococcus* growth efficiency. The above factors collectively promoted *Staphylococcus* to be the most dominant genus during microflora succession.

Additionally, differential metabolites such as 2,6-diaminopimelic acid, carveol, NAD^+^, coumaric acid, 3-succinoyl pyridine, and 2-hydroxycinnamic acid were positively correlated with *Bacillus* and *Corynebacterium* (*p* < 0.05; Figure 7). Carveol was a monocyclic monoterpene with a light mint aroma, coumaric acid and 2-hydroxycinnamic acid were the precursors of flavonoids, and 3-succinoyl pyridine was the nicotine degradation product (Huang et al., 2020). The results identified the positive roles of *Bacillus* and *Corynebacterium* in aroma substance formation. Additionally, the addition of inoculants enhanced the contribution rate of nicotinate and nicotinamide metabolism by *Corynebacterium* to 50.49% in BA_2 (Figure 4) and improved the *Corynebacterium* abundance in BA_3 to 21.29%, which was 31 times higher than that of CK_3 (0.68%; Figure 2B). The results suggested that under the addition of inoculants (BA group), nicotine degradation-associated NAD^+^ synthesis (Yu et al., 2015; Li et al., 2017; Li et al., 2021), NAD^+^/NADH redox equilibrium, and NADH-involved energy metabolism in *Corynebacterium* were more advantageous than that in *Staphylococcus*. Accordingly, the contribution rate of nicotine degradation and NAD^+^ anabolism by *Staphylococcus* in CK_2 was significantly decreased by 57% compared with the inoculant group (BA_2; Figure 4), identifying the positive roles of *Corynebacterium* in nicotine degradation for the first time.

Meanwhile, *Bacillus* and *Corynebacterium* were significantly negatively correlated with metabolites such as citric acid, fumaric acid, aconitic acid, GlcNAc-1-P, UDP-GlcNAc, and inositol (*p* < 0.05), and the *Bacillus* abundance in the microflora community of BA_3 was 3.96% (Figure 2B). The results indicated that *Bacillus* was in a disadvantageous state in the microflora succession and reflected a metabolic complementation between *Bacillus*, *Corynebacterium*, and *Staphylococcus* in the dynamically balanced fermentation ecosystem.

## 4 Conclusion

The effects of *B. altitudinis* inoculants on the microbial community diversity, shifts in the metabolism and differential metabolites, and the aroma substance production during CTL fermentation were studied. The addition of inoculants reinforced the CTL macromolecule transformation and facilitated the aroma production efficiently, and the total aroma production was increased by 43% compared with natural fermentation.

The omics analysis showed that *Staphylococcus* was the main contributor to fatty acid degradation, inositol phosphate metabolism, energy supply (oxidative phosphorylation), nutrient transport (ABC transporter and PTS), and aroma production (terpenoid backbone biosynthesis, phenylalanine metabolism, and degradation of aromatic compounds). Furthermore, *Staphylococcus* was positively correlated with TCA cycle intermediates (citric acid, fumaric acid, and aconitic acid), cell wall component–peptidoglycan intermediates (GlcNAc-1-P and UDP-GlcNAc), and phytic acid degradation products (inositol). The characteristics collectively facilitated *Staphylococcus* to be most dominant in the microbial community at the genus level during microflora succession. Accordingly, the relative abundance of *Staphylococcus* was increased from 2.94% (CK_0) to 93.11% (CK_3). Although the dominant genus was still *Staphylococcus* in BA_3 with an abundance of 53.69%, it was 42.4% lower than that of the control (CK_3), and the results suggested that the addition of inoculants supplemented the nutritional components of the CTLs, enhanced the metabolic activity and diversity of the bacteria such as *Corynebacterium*, improved their competitive advantages in the microflora succession, and facilitated the richness of microbial communities. Furthermore, the abundance of *Corynebacterium* was 21.29% (BA_3), which was 30.3-fold higher than that of the control (0.68%, CK_3).

The omics analysis also showed that although phosphate and *Bacillus altitudinis* that can dissolve the insoluble phosphate were added to the system, a phosphate-limited fermentation state seemed not to be resolved, and microflora demands on the soluble phosphate could be partially met by the phytic acid degradation dominated by *Staphylococcus*. Additionally, the addition of inoculants also improved nicotine degradation efficiency and enhanced its conversion to nicotinate, nicotinamide, and NAD^+^, which was mainly accomplished by *Corynebacterium* other than *Staphylococcus* during fermentation. Meanwhile, the significant correlation of differential metabolites with *Staphylococcus* and *Corynebacterium* showed that there was a metabolic complementation between them, thus forming a completely dynamic fermentation ecosystem.

The results provided further evidence for CTL fermentation optimization, such as the development of *Corynebacterium* inoculants and phosphate addition.

## Data Availability

The datasets presented in this study can be found in online repositories. The names of the repository/repositories and accession number(s) can be found in the article/Supplementary Material.
